# Effects of exergame training combined with omega-3 fatty acids on the elderly brain: a randomized double-blind placebo-controlled trial

**DOI:** 10.1186/s12877-019-1084-4

**Published:** 2019-03-13

**Authors:** Alexandra Schättin, Corinne Baier, Domenique Mai, Verena Klamroth-Marganska, Isabelle Herter-Aeberli, Eling D. de Bruin

**Affiliations:** 1 Department of Health Sciences and Technology, Institute of Human Movement Sciences and Sport, ETH Zurich, HCP, Leopold-Ruzicka-Weg 4, 8093 Zurich, Switzerland; 2 School of Health Professions, Zurich University of Applied Sciences, Winterthur, Switzerland; 3 Department of Health Sciences and Technology, Laboratory of Human Nutrition, ETH Zurich, LFV D22, Schmelzbergstrasse 7, 8092 Zurich, Switzerland; 4 Department of Neurobiology, Care Sciences and Society, Karolinska Institutet, Alfred Nobels Alle 23, 141 83 Huddinge, Sweden

**Keywords:** Nutritional supplementation, Omega-3 fatty acids, Physical exercise, Exergame, Brain function, Brain structure, Older adults, Aging

## Abstract

**Background:**

Older adults often suffer from age- and behavior-related brain changes affecting neuronal functioning and, therefore, cognitive and motor functions. The improvement of these functions might decrease falls and improve mobility. Previous studies indicate that video game-based physical exercise, so-called exergames, or omega-3 fatty acids (FAs) improve motor and cognitive functioning through brain adaptations. The aim of this study was to assess the effects of exergame training combined with fish oil supplementation on neuronal system levels in the brain and behavioral measurements in older adults. We hypothesized that the combination would differently affect these factors compared to the sole administration of exergame.

**Methods:**

Fifty-eight participants were randomly assigned to one of two groups (*N* = 29 each group) in a parallel, double-blind, randomized controlled trial lasting 26 weeks. The experimental group received daily fish oil, whereas the control group received daily olive oil. After 16 weeks, both groups started with an exergame training. Measurements were performed pre, during, and post intervention. Primary outcomes were recruitment curves using transcranial magnetic stimulation and response-locked potentials using electroencephalography. Secondary outcomes included executive functions and gait parameters. Blood samples were taken to control for FAs.

**Results:**

Forty-three individuals (mean age 69.4 ± 4.6 years) completed the study (N_experimental_ = 22, N_control_ = 21). The results showed no significant time × group interaction effects for any parameters. Blood samples demonstrated significant time × group interaction effects. Post-hoc tests showed a significant increase of omega-3 FAs (*p* < .001) and a significant decrease of omega-6 FAs (p < .001) for the experimental group.

**Conclusion:**

The combination of exergame training and fish oil did not lead to additional beneficial effects. To trigger possible effects, future studies should carefully consider study design aspects; e.g. study duration, individual nutritional supplementation dose, omega-3 FAs supplementation composition, and placebo. Furthermore, studies should consider neuroimaging methods as these might be more sensitive to assess early brain adaptations. Thus, future studies should be aware of several aspects running a combinatory study that includes omega-3 FAs according to their expected effects.

**Trial registration:**

Swiss National Clinical Trials SNCTP000001623 and ISRCTN12084831 registered 30 November 2015.

## Background

Age- and behavior-related changes in the human brain involve structural, functional, and metabolic levels. Age- and behavior-associated alterations in white matter integrity, grey matter volume [1–3], and neurotransmitter (e.g. dopamine, serotonin, and acetylcholine) synthesis and binding [4–8] go along with deteriorations of cognitive functions, e.g. executive functions (EFs). EFs are higher-level cognitive functions that control and guide lower-level cognitive functions and goal-directed actions [9], such as walking in challenging environments. Gait performance is partially controlled by different EF components, e.g. “working memory” [10], “inhibition” [11], and “divided attention” [12]. Especially divided attention is associated with temporal and spatial dual-task gait parameters [13]. Gait disturbances and falls seem to be related to the quality of EFs [14, 15].

The prefrontal cortex (PFC), especially the dorsolateral prefrontal cortex and connected brain structures, has been linked with EFs [16, 17]. Better EFs are associated with a greater PFC thickness and a larger PFC volume [18]. During life, the (pre)frontal structure undergoes transformation processes; however, no agreement exists on the specific pattern of EFs adaptations [1, 17, 19, 20]. One presumption is that a decrease in frontal grey matter volume and white matter integrity might be related to a decline of EFs. Rosano et al. (2012) illustrated that a smaller PFC volume might contribute to slower gait performance due to decreased information processing capacity [21]. Furthermore, disrupted communication of cortico-cortical and cortico-subcortical networks, e.g. connection of frontal parts with parietal lobe and basal ganglia, respectively, are common causes of higher-level gait disorders [15, 22]. Consequently, strengthening of EFs might improve gait performance [23] and concomitantly might reduce falls as the risk of future falls can be predicted by EFs performance in older adults [24].

Up to now, training of specific cognitive functions (e.g. EFs) may represent a central method to support specific brain functions and also preserve mobility in older adults [21, 25–27]. Nonetheless, recent reviews examining the interaction of cognitive and physical functions concluded that a combined motor-cognitive training seems to be important for clinical practice to achieve safe movements in daily environment [27–30]. On neuronal level, physical training triggers brain plasticity by cell proliferation and synaptic plasticity, while cognitive training seems to support the survival of newborn neurons and their integration in pre-existing networks [28, 31, 32]. Especially, computerized training interventions seem effective [27, 28, 33] when providing training principles that support (motor) learning [33]. Video game-based physical exercises, or so-called exergames, allow concurrent training of motor and cognitive abilities. Incorporated video games are promising to train various cognitive functions [34, 35]. Physical exercise (PE) interventions with decision-making opportunities are potentially able to improve both motor performance and cognition [36]. Recent studies showed positive effects of exergame training on EFs and gait performance under dual-task condition in older adults [37, 38] and a meta-analysis revealed that both healthy older adults and clinical populations with conditions associated with neurocognitive impairments benefit from physical-active video games [39].

In various review articles, it is hypothesised that the impact of PE on the brain can be supported by concurrent intake of specific nutrients [40–44]. This would mean, as a way of example, that a combination of PE with a nutritional supplement (NS) might further intensify the effects of PE on brain structure and function in older adults. The possible interplay between PE and nutrition involves common cellular processes essential for synaptic plasticity, neurogenesis, cell survival, and vascular function [40–44].

Nonetheless, a recent systematic review concluded that former studies executing a combined approach of PE and NS to evoke neuronal adaptations were not particularly successful due to the misfit between the combinations; the elements were not chosen based on sharing of similar neuronal mechnism [45]. The review argues, however, that especially omega-3 fatty acids (FAs), present in fish oil, might be an efficient NS promoting the beneficial effects of PE. Omega-3 FAs are essential for energy metabolism, for the function and integrity of the neuronal plasma membranes (with docosahexaenoic acid (DHA), arachidonic acid, and eicosapentaenoic acid (EPA) as their main components), and for blood perfusion in the brain [46, 47]. Particularly, older adults may profit from FA supplementation, as in the aging brain the concentration of long chain polyunsaturated FAs (LCPUFAs) concentration decreases [46]. LCPUFAs intake improves cognition, decreases (neuro)inflammation, and reduces vascular risk factors in older adults [46]. On brain level, LCPUFAs may have positive effects on neuronal structure, function, and cerebral blood flow [48]. For example, DHA acts as a neurotrophic factor by increasing the level of the brain-derived neurotrophic factor [49]. Previous randomized-controlled studies showed that fish oil enhanced brain structure and function in healthy older adults, and participants improved working memory, EFs, white matter microstructure integrity, grey matter volume, and vascular parameters [50, 51].

So far, studies could show that DHA supplementation enhanced the effects of exercise on axonal growth, brain derived neurotrophic factor-related synaptic plasticity, and cognition in rats [49, 52]. However, no study exists that examined the combined effect of exergame training and omega-3 FAs on the brain in healthy older adults. This study, therefore, aims to investigate whether the positive effects of exergame training can be enhanced through adding omega-3 FA supplementation. The following research question guided the research process: “Does the combination of exergame training and fish oil differently affect neuronal system levels in the elderly brain compared to exergame training alone?” The main objectives of this study were to determine the effects of the intervention on corticospinal excitability and neuronal activity. We hypothesized that the combination would differently affect these parameters.

## Methods

### Study design

The study was a randomized double-blinded, placebo-controlled trial involving older adults above 65 years. Detailed information about the study procedure and protocol has been previously described [53]. Between December 2015 and June 2016, potential participants were recruited from the Senior’s University Zurich (Switzerland), senior residency dwellings in Zurich (Switzerland), and through public advertisement and flyers. The study was divided in three blocks, the first block started at the beginning of March 2016, the second block at the beginning of May 2016, and the third at the beginning of June 2016. Measurements and data collection, exergame training, and data analysis were performed at the same study site (ETH Hönggerberg, Zurich, Switzerland). The participants were provided with NS for home supplementation and expected to take the NS regularly. Measurements were performed before and after the intervention period of 26 weeks. For blood samples, an additional measurement was performed before the training started (after 16 weeks). The local ethics committee (EC Zurich Switzerland, EC number: 2015–0190) approved the study procedure. The study procedure conforms to the Declaration of Helsinki and the guidelines of Good Clinical Practice E6 (R1). Written informed consent was given by the participants before any data was recorded. This study has been registered in the Swiss National Clinical Trials (SNCTP000001623) and the ISRCTN (ISRCTN12084831) portals. The study followed the Consolidated Standards of Reporting Trials (CONSORT) statement on randomized trials of non-pharmacological treatment [54].

### Participants

Participants fulfilling all of the following inclusion criteria were eligible to partake: (1) age at or above 65 years, (2) live independently or in a senior residency dwelling, (3) healthy (self-reported), and (4) non-smoker. Participants were excluded when they showed one of the following exclusion criteria: (1) mobility impairments that prevent training execution, (2) rapidly progressive or terminal illness as well as acute (time frame of 3 to 14 days, e.g. flu) or chronic illness (where “chronic” is something that is “continuing or occurring again and again for a long time.” [55]), (3) orthopedic or neurological diseases (e.g. stroke or epilepsy) that prevent training participation, (4) history of heart attack, (5) medication that interacts with NS (i.e., oral hypoglycemic drugs or anticoagulants), (6) medication that acts on neuronal level (i.e., psychotropic medications), (7) cognitive impairment (Mini Mental Status Examination < 22 points) [56], (8) signs of depression (Geriatric Depression Scale > 5 points), (9) electronic or metallic head implants (self-reported), and (10) personal history of dizziness (self-reported). Prior power calculation can be found in the previously published study protocol [53].

### Interventions

The study intervention has been previously published in detail [53] in line with the Template for Intervention Description and Replication (TIDieR) guidelines [57].

### Nutritional supplementation

The NS were packed in dark-glass bottles (200 ml) identical in outer appearance. An external center (Kantonsapotheke Zurich, Switzerland) was responsible for blinding to achieve double blinding. For group randomization, a computer-generated list including numbers from 001 to 060 was created by an external center (for detailed information [53]). The individual who maintained the master randomization list (EDB) was responsible for assigning randomization codes, securely storing all randomization files out of reach and sight of the other investigators, and notifying appropriate study staff that the participant had been randomized. The groups were referred to without specification of NS (e.g., group A and B) for statistical analysis.

In the experimental group, the participants consumed liquid fish oil (San Omega GmbH, Akersbakken 35B, NO-0172 Oslo). In the control group, the participants consumed the same amount of olive oil as placebo (Oro del Desierto, Ctra. Nacional 340, 04200 Tabernas, Almeria, Spain). Olive oil as placebo was selected because of its similarity in color, consistency, taste, and composition and because it is the most frequently used comparator for omega-FA studies [58]. Furthermore, the participants had no direct comparison of the NS to support the blinding situation. Over 26 weeks, the participants consumed a daily amount of 13.5 ml of fish oil per day, including 2956.5 mg of omega-3 FAs (1471.5 mg EPA and 162 mg DHA), or 13.5 ml of olive oil per day. The NS could be either taken undiluted or could be added to food (e.g. salads) or drinks at home. On the bottles, a label indicated that unopened bottles can be stored at room temperature (15–25 °C) protected from direct sun light. The opened bottle has to be stored in the refrigerator (2–8 °C) and used within 45 days. A measuring cup was used to standardize and control the intake amount of 13.5 ml. An intake rate of less than 80% was set as an exclusion criterion for the analysis. An NS diary was supplied to check for intake adherence. Moreover, the participants were instructed to continue with their normal nutritional habits during the intervention.

### Exergame training

The participants performed specific whole-body movements, especially step executions, on a pressure-sensitive plate (Impact Dance Platform, 87.5 cm × 87.5 cm × 2.5 cm, Positive Gaming BV, BZ Haarlem, Nederland) to interact with the video game (VG) presented on a frontal computer desktop. The VG (Dividat AG, Schindellegi, Switzerland) were designed to specifically trigger the use of EFs. More information about implemented feedback mechanism and training principles can be found in the study protocol [53].

After 16 weeks, all participants started to perform the exergame training lasting 10 weeks. The participants trained three times per week, where one session took 30 min. Exergame training contained six different VG (each 4 min) in a predefined order and with short breaks (~ 1 min) for game change. In training rooms, the participants performed their exercises alone or in small groups of two to four and were supervised by experienced investigators. To check for adherence, the participants received a training plan and a checklist was used by the investigators.

### Primary outcomes

#### Transcranial magnetic stimulation

On an adjustable chair, participants were comfortably seated with hip, knee, and ankle joint angles of 100°, 120°, and 90°, respectively. Single pulse cortical stimulation was applied by means of a transcranial magnetic stimulation (TMS) stimulator MAGSTIM 200 (Magstim Company Ltd., Whitland, Dyfed, UK) with a “figure of eight” coil placed over the cortical motor area to stimulate the right *M. tibialis* anterior (TA). Only the dominant side was assessed because of the symmetrical nature of TMS-related measurements of the lower limb [59].

A recruitment curve (RC) of increasing intensities of 10% steps was obtained in ten trials per step. The stimuli intensities from 90% to 140% resting motor threshold (RMT) were applied in a random order [60]. Telemyo DTS (Noraxon Inc., Arizona, USA) system was used to record muscle activity. On the muscle belly of the right TA, two electrodes (Ambu® Blue Sensor N, Cambridgeshire, UK) were placed with an inter-distance of two centimeters. The analysis of peak-to-peak amplitude of motor evoked potential (MEP) was performed in Matlab™ for Windows (Mathworks Inc., Natick, MA, USA). The peak-to-peak amplitudes of each stimulation intensity were averaged and normalized to the individual maximal pre-measurement RMT (140%). The slope of the RC was calculated as the slope of the linear regression line through a supplied set of x-values (normalized MEP values) and y-values (stimulation intensities). For statistics, the MEPs of the different stimulation intensities and the slope of the RCs were included.

#### Electroencephalography

The participants wore the 20-channel dry-electrodes Enobio device (Neuroelectrics, Barcelona, Spain) [61, 62]. The device sent the data via Bluetooth connection to a personal computer where data were monitored in real-time using Neuroelectrics Instrument Controller 1.4.8 software. During data recording, the participants performed a Go/No-go task (Test for Attentional Performance (TAP; see following paragraph for company details)). On a personal computer screen, the task was presented in front of the participants for about 10 min (five times 2 min). The software randomly presented stimulus (×) or (+). The participant had to push a predefined button in the presence of the critical stimulus (×) and had to inhibit the push in the presence of the noncritical stimulus (+). One performance lasted 2 min and included 40 stimuli (20 critical and 20 noncritical stimuli) whereby each stimulus was presented for 200 ms followed by a silent period (black screen) of 2800 ms until the next stimulus appearance. The participants had to push a predefined button on a keyboard when the critical stimuli (×) appeared (N_Total_ = 100). One investigator recorded the right (×) and wrong (+) responses of the participants comparing the stimuli of the Go/No-go task and the trigger appearing on the EEG screen. A trigger was recorded at the time point of clicking and integrated into the EEG data. The trigger signals were used for further analysis of the EEG data including response-locked potentials (RLP). RLP allow the determination of neural processes involved in monitoring performance [63]. Studies indicated that chronic exercise and/or fitness are related to more efficient response monitoring and upregulation of cognitive control [63, 64]. For time reasons and due to technical difficulties, EEG analysis was focused on RLP and no EEG acquisition was performed during gait performance.

Off-line signal processing was performed using EEGLAB [65] and ERPLAB [66] toolboxes for Matlab™ for Windows (Mathworks Inc., Natick, MA, USA). Based on previous studies, the focus of analysis was set to prefrontal-located channels Fp1 and Fp2 as well as midline-located channels from frontal to parietal (Fz, Cz, and Pz) [38, 67, 68]. First, the five runs of the Go/No-go task were merged and divided by 1000 to build one dataset in μV for each participant. Then, dataset was filtered (zero phase FIR filters) using high-pass filter at 0.1 Hz, notch filter (48 Hz – 52 Hz), and low-pass filter at 30 Hz. Afterwards, bad channels were rejected using joint probability of the recorded electrodes (kurtosis above 5 standard deviations from the mean of all channels). The next step was re-referencing of the data to the average followed by deletion of events (clicks) appearing at “irrelevant” stimuli (No-go signals). Then, RLP were averaged for 1000 ms preceding the response onset and 1400 ms following it including baseline correction [− 1000 ms to -600 ms] according to Luck (2014) [69]. In addition, the epochs containing values exceeding ± 50 μV were rejected. The analysis resulted in repeatable RLP for the prefrontal located channels Fp1 and Fp2 but not for the midline-located channels (Fz, Cz, and Pz). Statistical analysis was performed focusing on channel Fp1 and Fp2. For Fp1 and Fp2, the focus was on peak amplitude for the positive peak before and for the negative peak after response onset.

### Secondary outcomes

#### Test for attentional performance

The TAP (D-TAP 2.3 VL, PSYTEST, Psychologische Testsysteme, Herzogenrath, Germany) was established to measure attention deficits. The TAP is a valid computer-based assessment including subtests that measure different and statistically independent attentional aspects [70]. Each test was played on a screen of a personal computer in front of the participants. To clarify the procedure and to minimize possible learning effects, the participants first performed a pre-test. The participants executed two tests: (1) Working memory test (5 min) and (2) Divided attention test (3.25 min). For each test, reaction time, errors, and omissions were recorded. Cognitive testing focused on the TAP test battery, while predefined Attention Network Test was discarded to prevent too high participant burden of testing.

#### Gait analysis

Spatial (distance) and temporal (time) gait parameters, including speed, cadence, stride length, and minimal toe clearance, were measured with the Physilog (Gait up Sàrl, Lausanne, Switzerland) via wearable standalone movement inertial sensors (50 × 37 × 9.2 mm, 19 g, anatomical curved shape). This gait analysis device provides quantitative, objective, and valid assessment of gait movement [71–74]. Elastic straps were used to fix the sensors at the right and left forefoot of the participants for flat over ground gait analysis over a distance of 10 m. Participants executed a single-task condition at preferred walking speed and a dual-task condition, i.e. preferred walking speed whilst counting backwards in steps of seven from a random given number between 200 and 250. For more information about the gait analysis procedure, we refer to the trial article [53]. The means of three successful trials were used for further analysis. In addition to spatio-temporal gait parameters, the relative dual-task cost (DTC) of walking, as percentage of loss relative to the single-task walking performance was calculated (DTC [%] = 100 * (single-task score – dual-task score)/ single-task score [75]). Acceleration and deceleration steps were removed from the data to analyze steady state walking.

### Control outcomes

#### Blood sample

A qualified investigator collected venous blood samples and stored the samples in 2.7 ml EDTA tubes (S-Monovette, K3 EDTA, 75 × 13 mm, Sarstedt, Germany). To analyze FA concentrations in erythrocytes, blood samples were taken at pre-, during- (after 16 weeks), and post-intervention. In this study, the focus was on the omega-3 FAs, EPA, DHA, omega-3 index, omega-6 FAs, and oleic acid within red blood cells. Omega-3 index is defined as the percentage of EPA and DHA in the red cell membrane, with the remaining FAs building up to 100% [76]. The FA parameters were analyzed using a standardized method (gas chromatograph) under rigorous quality control (DIN ISO 15189) by Omegametrix GmbH (Martinsired, Germany).

#### Cognitive status, depression, and fall efficacy scale

The Mini Mental State Examination (MMSE) is a valid and reliable test to quantitatively estimate the cognitive status [77, 78]. To identify depression in older adults, the Geriatric Depression Scale (GDS) is a reliable and valid self-report depression screening questionnaire [79, 80]. The short form Falls Efficacy Scale International (FES-I) is a feasible scale to determine fear of falling in older adults [81].

### Statistics

All statistical procedures were conducted with the IBM Statistical Package for the Social Science software package, version 22. A per protocol analysis was performed. Data were tested for normal distribution using Shapiro-Wilk test and Q-Q-plots. Baseline comparisons were undertaken using Mann-Whitney U test. Due to non-normal distribution, the data were rank-ordered in order to perform, for each variable, a repeated measures analysis of variance with one within-subjects factor (time: pre-, (during-), and post-measurement) and one between-subjects factor (intervention group: fish oil and exergame/ olive oil and exergame). The analysis allowed to compare time × group interaction and time main effects, using the Puri and Sen *L* Statistics for ranked data [82]. *L* value was calculated using Pillai’s Trace:

$$ L=\left(N-1\right){r}^2 $$

where N = amount of participants and r^2^ = Pillai’s Trace. Within-group comparisons for significant interaction effects were performed using Friedman test and post-hoc Dunn-Bonferroni test. A probability level of *p* <  .05 was considered to be statistically significant. Effect sizes assessing meaningfulness of differences within- and between-group design were calculated and expressed using the following equation:

$$ \mathrm{r}=\frac{\left|Z\right|}{\sqrt{N}} $$

where Z = Z-score and N = amount of participants. An effect size of r = 0.1 is considered a “small” effect, around 0.3 a “medium” effect, and 0.5 and above a “large” effect. Effect sizes assessing between × within group design were calculated and expressed as η_p_^2^ and η_G_^2^ and where percentage of the total variance can be accounted for by group membership [83], using the following equations:

$$ {\upeta}_p^2=\frac{SS_{effect}}{SS_{effect}+{SS}_{error}} $$

$$ {\upeta}_G^2=\frac{SS_{effect}}{SS_{effect}+{SS}_{error\ between}+{SS}_{error\ within}} $$

where SS = sum of squares.

## Results

**Fig. 1:**
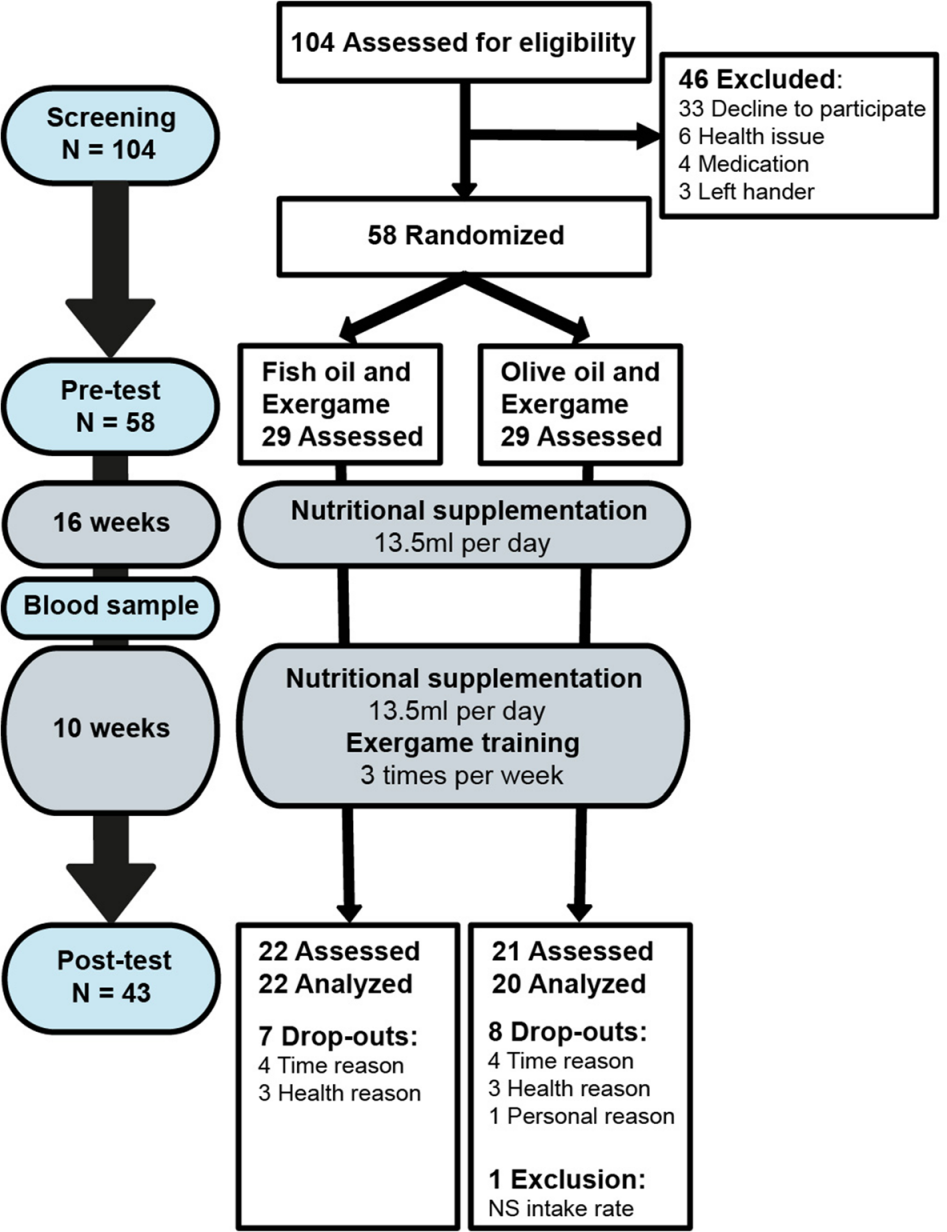
Study flow chart. NS = nutritional supplementation

A total of 58 participants were randomly assigned to one of the two groups: (1) fish oil and exergame training or (2) olive oil and exergame training. Forty-three participants completed the whole study procedure. One participant was excluded from analysis because the supplementation intake was 39%. The study flow chart is presented in Fig. 1. The analysis does not consider intention-to-treat analysis because of a clear description of the reason(s) for drop-out (CONSORT 2010 guidelines [84]). Table 1 summarizes demographic characteristics, screening values, and intervention details of the participants. The significant difference for the GDS value was not considered for the analysis, since all the values were in the normal range indicating no signs of depression. No differences were found for the baseline characteristics between the 58 included participants and the 42 analyzed participants. All the participants reached a minimal amount of 70% (21 training sessions) training participation.

**Table 1:** Demographic characteristics, screening values, and intervention details

	Fish oil and Exergame (*N* = 22)	Olive oil and Exergame (N = 20)	z	p	r
Baseline					
Gender [F/M]	[10/ 12]	[13/ 7]			
Age [years]	67 (65.75; 72.50)	67.50 (65.25; 75.75)	−0.537	.592	0.08
Weight [kg]	76.5 (62.5; 85.25)	74.50 (61.75; 81.50)	−0.554	.579	0.09
Height [m]	1.70 (1.62; 1.80)	1.69 (1.60; 1.76)	−1.148	.251	0.18
Body Mass Index	25.66 (22.80; 27.32)	25.49 (23.47; 27.93)	−0.302	.762	0.05
Mini Mental Status	28.5 (27.75; 29.25)	28 (28; 29)	−0.495	.620	0.08
Geriatric Depression Scale	1 (0; 3)	0 (0; 1)	−2.209	.027^*^	0.34
Short-FES-I	8 (7; 9.25)	7 (7; 8.75)	−1.203	.229	0.19
Resting motor threshold	42 (40; 45)	43.5 (38.5; 50)	−0.540	.589	0.13
Intervention					
Absolved trainings (100% = 30 sessions)	27 (23; 29)	26 (22.25; 27)	−0.881	.378	0.14
Supplementation intake [%]	98.2 (94.5; 100)	96.4 (91.8; 99.5)	−0.840	.401	0.13

Data are number of participants or median (interquartile range) values as indicated. *p*-values were calculated using Mann-Whitney U test. *p < .05. For effect size r; r = 0.1 is considered a small effect, around 0.3 a medium effect, and 0.5 and above a large effect. *FES-I* Falls efficacy scale international

### Motor evoked potentials

**Fig. 2:**
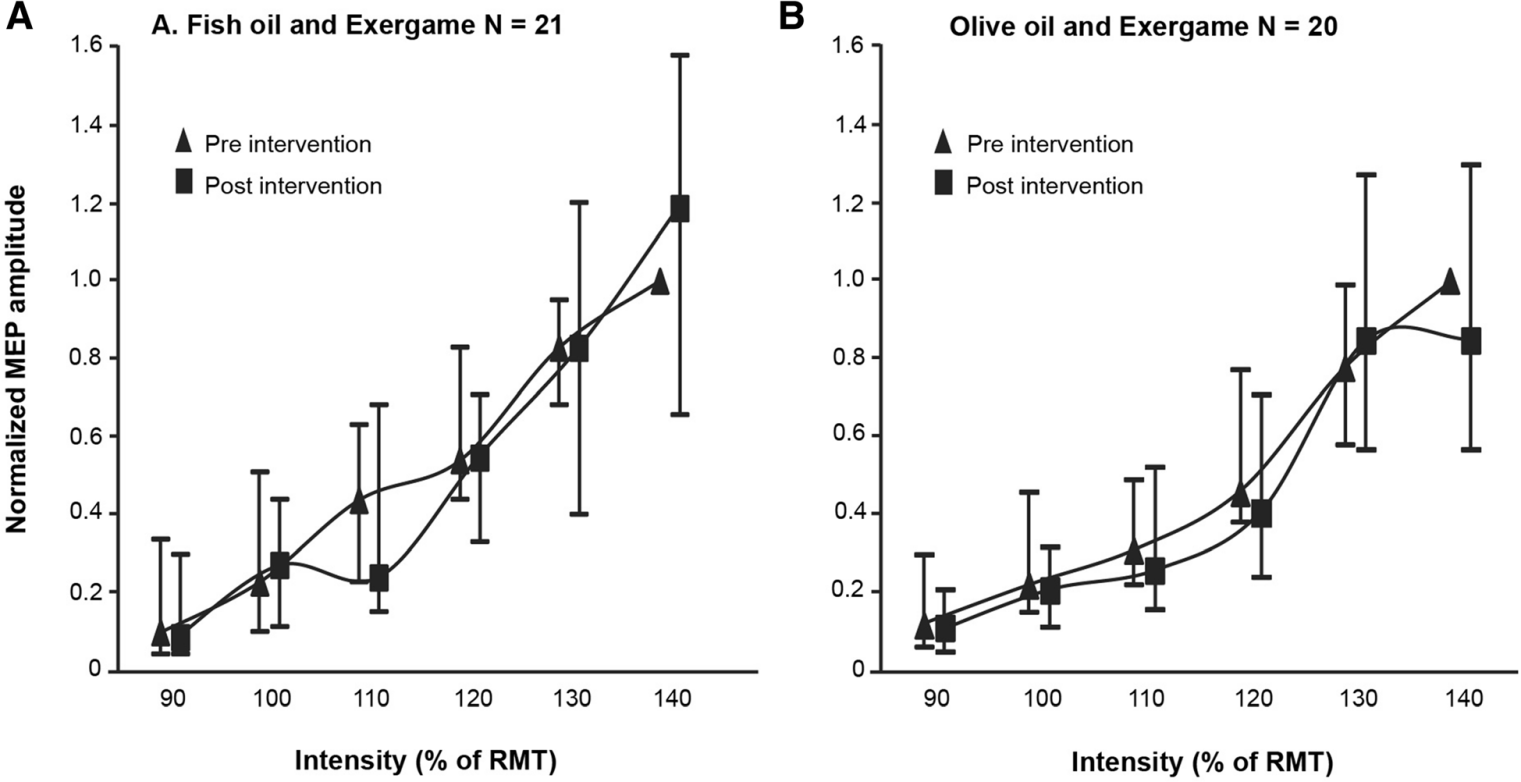
Pre- and post-measurement values of motor evoked potential (MEP) building the recruitment curve. Data are median values (interquartile ranges) as indicated. MEP values were normalized to the individual maximum (140%) pre-measurement resting motor threshold (RMT)

For the analysis, one participant had to be excluded due to a technical problem. The RCs including pre and post MEPs for both groups are illustrated in Fig. 2. Table 2 summarizes the time × group interaction and time main effects for MEPs and RC slopes. No significant time × group interaction effects were present.

**Table 2:** Time × group interaction and time main effects of repeated measures Puri & Sen-analysis of ranked data for motor evoked potentials and recruitment curve slopes

	Interaction effect (time × intervention)					Main effect (time, pre vs. post)				
	r^2^	L	p	Ƞ_p_^2^	Ƞ_G_^2^	r^2^	L	p	Ƞ_p_^2^	Ƞ_G_^2^
Stimulation intensity										
90% RTM	0.002	0.084	.776	< 0.01	< 0.01	0.021	0.831	.369	0.02	< 0.01
100% RTM	0.015	0.611	.441	0.02	< 0.01	0.015	0.611	.441	0.02	< 0.01
110% RTM	0.019	0.745	.395	0.02	< 0.01	0.125	4.993	.023^*^	0.12	0.02
120% RTM	0.011	0.428	.520	0.01	< 0.01	0.091	3.623	.056	0.09	0.02
130% RTM	0.001	0.026	.875	< 0.01	< 0.01	0.000	0.002	.963	< 0.01	< 0.01
140% RTM	0.038	1.522	.222	0.04	0.02	0.018	0.730	.400	0.02	0.01
Recruitment curve slope	0.027	1.080	.307	0.03	0.01	0.010	0.400	.307	0.01	< 0.01

*N* = 41; fish oil and exergame group *N* = 21 and olive oil and exergame group *N* = 20. *p < .05. η_p_^2^: effect size. η_G_^2^: effect size

### Response-locked potentials

For the analysis of Fp1, ten participants had to be removed because no RLP was present. For the analysis of Fp2, data from two participants had to be removed because of channel artifacts and data from seven participants had to be removed because no RLP was present. The RLP of Fp1 and Fp2 showed an equal composition with a positive peak before the response onset followed by a negative peak after the response onset. The total number of epochs, used for analysis, were for the fish oil and exergame group pre (Fp1: *N* = 1838, Fp2: *N* = 1868), post (Fp1: *N* = 1626, Fp2: *N* = 1650) and for the olive oil and exergame group pre (Fp1: *N* = 1499, Fp2: *N* = 1500), post (Fp1: *N* = 1542, Fp2: *N* = 1463). Response-locked potentials for Fp1 and Fp2 of both groups (pre and post) are presented in Table 3. Table 4 summarizes the time × group interaction and time main effects for RLP in Fp1 and Fp2. No significant time × group interaction effects were present; neither for Fp1 nor for Fp2.

**Table 3:** Pre- and post-measurement values of response-locked potentials for Fp1 and Fp2

	Fish oil and Exergame		Olive oil and Exergame	
	Pre (N = 20)	Post (*N* = 19)	Pre (*N* = 17)	Post (N = 17)
Positive peak before response onset [μV]				
Fp1	1.41 (1.14; 2.72)	1.89 (0.94; 3.78)	1.58 (1.14; 2.50)	2.90 (1.27; 4.07)
Fp2	1.55 (1.07; 2.31)	1.64 (1.12; 3.19)	1.81 (0.97; 2.12)	2.95 (1.10; 3.90)^1^
Negative peak after response onset [μV]				
Fp1	−3.43 (−5.45; −1.61)	−4.14 (−6.68; −1.47)	−2.36 (−4.66; −1.45)	−4.43 (−6.70; −2.20)
Fp2	−2.84 (−4.89; − 1.53)	−3.64 (− 5.40; − 1.82)	−2.60 (− 4.93; − 1.78)	−5.17 (− 6.55; − 1.86)^1^

Data are median values (interquartile ranges) as indicated. ^1^ N = 16

**Table 4:** Time × group interaction and time main effects of repeated measures Puri & Sen-analysis of ranked data for response-locked potentials

	Interaction effect (time × intervention)					Main effect (time, pre vs. post)				
	r^2^	L	p	Ƞ_p_^2^	Ƞ_G_^2^	r^2^	L	p	Ƞ_p_^2^	Ƞ_G_^2^
Positive peak before response onset										
Fp1	0.001	0.031	.863	< 0.01	< 0.01	0.127	3.922	.046*	0.13	0.03
Fp2	0.018	0.572	.458	0.02	< 0.01	0.189	6.054	.011*	0.19	0.04
Negative peak after response onset										
Fp1	< 0.001	< 0.001	.995	< 0.01	< 0.01	0.123	3.816	.049*	0.12	0.03
Fp2	0.022	0.698	.412	0.02	< 0.01	0.137	4.393	.034*	0.14	0.03

For Fp1 *N* = 32; fish oil and exergame group N = 17 and olive oil and exergame group N = 15. For Fp2 *N* = 33; fish oil and exergame group N = 18 and olive oil and exergame group *N* = 15. *p < .05. η_p_^2^: effect size. η_G_^2^: effect size

### Executive functions

For working memory and divided attention test, the reaction time, errors, and omissions of both groups (pre and post) are presented in Table 5. Table 6 summarizes the time × group interaction and time main effects for working memory and divided attention test. No significant time × group interaction effects were present.

**Table 5:** Pre- and post-measurement values of working memory and divided attention test

	Fish oil and Exergame (N = 22)		Olive oil and Exergame (N = 20)	
	Pre	Post	Pre	Post
Working memory				
RT [ms]	688.50 (587.75; 811)	725 (599; 758)	658.5 (589.75; 863)	748 (611.50; 838.25)
Errors	1 (0; 2.25)	1 (0; 3)	2.5 (1; 5)	2 (1; 5.5)
Omissions	1 (0; 3.25)	1.5 (0; 3)	3 (2; 5)	2 (1; 4.5)
Divided attention				
RT auditory [ms]	649.50 (554.50; 712)	684 (611.25; 729)	674.50 (615.50; 759)	691 (629; 764.50)
RT visual [ms]	950 (838.75; 988.75)	927.5 (831.5; 1015.25)	895.5 (830.75; 1000.75)	936 (825.25; 1018)
Errors	2 (1; 5.5)	1 (0; 1.25)	2 (0; 5.5)	1 (0; 2)
Omissions	1.5 (1; 4)	1.5 (0; 3)	2.5 (2; 4.75)	2 (1; 3)

Data are median values (interquartile ranges) as indicated. *RT* Reaction time

**Table 6:** Time × group interaction and time main effects of repeated measures Puri & Sen-analysis of ranked data for working memory and divided attention test

	Interaction effect (time × intervention)					Main effect (time, pre vs. post)				
	r^2^	L	p	Ƞ_p_^2^	Ƞ_G_^2^	r^2^	L	p	Ƞ_p_^2^	Ƞ_G_^2^
Working memory										
Reaction time	0.047	1.930	.168	0.05	0.01	0.040	1.659	.202	0.04	0.01
Errors	0.020	0.818	.372	0.02	< 0.01	0.003	0.137	.716	< 0.01	< 0.01
Omissions	0.029	1.201	.280	0.03	0.01	0.027	1.095	.301	0.03	0.01
Divided attention										
Reaction time auditory	0.003	0.126	.727	< 0.01	< 0.01	0.067	2.727	.099	0.07	0.01
Reaction time visual	0.011	0.447	.511	0.01	< 0.01	0.014	0.586	.451	0.01	< 0.01
Errors	0.056	2.312	.130	0.06	0.02	0.334	13.700	<.001^*^	0.33	0.14
Omissions	0.006	0.257	.618	0.01	< 0.01	0.054	2.227	.137	0.05	0.02

*N* = 42; fish oil and exergame N = 22 and olive oil and exergame N = 20. *p < .05. η_p_^2^: effect size. η_G_^2^: effect size

### Spatio-temporal gait parameters

For the toe clearance, dual-task walking and DTC analysis, one participant had to be excluded due to a technical problem. Spatio-temporal gait parameters including speed, cadence, stride length, and toe clearance of both groups (pre and post) are presented in Table 7. Table 8 summarizes the time × group interaction and time main effects for spatio-temporal gait parameters. No significant time × group interaction effects were present.

**Table 7:** Pre- and post-measurement values of spatio-temporal gait parameters

	Fish oil and Exergame (*N* = 22)		Olive oil and Exergame (*N* = 20)	
	Pre	Post	Pre	Post
Speed [m/s]				
Single-task	1.34 (1.29; 1.42)	1.36 (1.23; 1.47)	1.33 (1.25; 1.45)	1.36 (1.24; 1.45)
Dual-task	1.15 (0.96; 1.24)	1.22 (1.11; 1.28)	1.12 (0.94; 1.23)	1.17 (0.95; 1.28)
Dual-task cost [%]	14.01 (7.52; 24.72)	11.91 (4.16; 16.70)	13.51 (9.91; 23.61)	10.83 (7.00; 23.86)
Cadence [steps/min]				
Single-task	112.82 (109.41; 118.39)	113.92 (110.48; 122.12)	116.93 (109.24; 121.82)	118.43 (106.95; 122.09)
Dual-task	102.50 (97.03; 112.58)	109.15 (100.21; 112.97)	104.85 (88.10; 114.56)	106.57 (89.53; 119.31)
Dual-task cost [%]	7.16 (2.68; 12.18)	5.74 (1.48; 9.19)	10.71 (4.56; 16.59)	6.61 (2.39; 14.52)
Stride length [m]				
Single-task	1.42 (1.33; 1.48)	1.43 (1.36; 1.48)	1.40 (1.27; 1.44)	1.37 (1.33; 1.45)
Dual-task	1.31 (1.23; 1.42)	1.35 (1.27; 1.43)	1.29 (1.18; 1.37)	1.30 (1.20; 1.39)
Dual-task cost [%]	7.40 (3.93; 11.67)	4.96 (2.80; 8.88)	6.70 (4.12; 9.72)	5.40 (2.81; 8.80)
Minimal toe clearance [m]				
Single-task	0.025 (0.021; 0.028)	0.028 (0.023; 0.033)	0.026 (0.019; 0.031)	0.029 (0.023; 0.035)
Dual-task	0.025 (0.021; 0.028)	0.027 (0.022; 0.032)	0.024 (0.021; 0.029)	0.029 (0.028; 0.035)^1^
Dual-task cost [%]	0.48 (−9.49; 6.07)	5.23 (−16.11; 13.29)	8.64 (−3.28; 14.53)	0.94 (−10.45; 6.59)^1^

Data are median values (interquartile ranges) as indicated. ^1^N = 19

**Table 8:** Time × group interaction and time main effects of repeated measures Puri & Sen-analysis of ranked data for spatio-temporal gait parameters

	Interaction effect (time × intervention)					Main effect (time, pre vs. post)				
	r^2^	L	p	Ƞ_p_^2^	Ƞ_G_^2^	r^2^	L	p	Ƞ_p_^2^	Ƞ_G_^2^
Speed										
Single-task	< 0.001	0.012	.915	< 0.01	< 0.01	0.002	0.068	.798	< 0.01	< 0.01
Dual-task	0.005	0.214	.649	0.01	< 0.01	0.151	6.182	.011^*^	0.15	0.02
Dual-task cost	< 0.001	0.013	.910	< 0.01	< 0.01	0.161	6.603	.008^*^	0.16	0.04
Cadence										
Single-task	0.010	0.419	.524	0.01	< 0.01	0.013	0.521	.477	0.01	< 0.01
Dual-task	< 0.001	0.018	.895	< 0.01	< 0.01	0.102	4.179	.039^*^	0.10	0.02
Dual-task cost	0.002	0.084	.775	< 0.01	< 0.01	0.124	5.078	.022^*^	0.12	0.03
Stride length										
Single task	0.003	0.103	.753	< 0.01	< 0.01	0.002	0.085	.774	< 0.01	< 0.01
Dual-task	0.010	0.393	.537	0.01	< 0.01	0.177	7.250	.006^*^	0.18	0.01
Dual-task cost	0.008	0.344	.564	0.01	< 0.01	0.123	5.036	.023^*^	0.12	0.02
Toe clearance										
Single-task	0.005	0.189	.669	< 0.01	< 0.01	0.160	6.551	.009^*^	0.16	0.04
Dual-task^1^	0.027	1.122	.301	0.03	0.01	0.177	7.252	.006^*^	0.18	0.06
Dual-task cost ^1^	0.071	2.928	.091	0.07	0.03	0.004	0.154	.703	< 0.01	< 0.01

N = 42; fish oil and exergame N = 22 and olive oil and exergame N = 20. *p < .05. η_p_^2^: effect size. η_G_^2^: effect size. ^1^N = 41

### Blood fatty acids level

The blood values of both groups (pre, middle, and post) are presented in Table 10. Significant time × group interaction effects were present for Omega-3 index (L(41) = 27.349, *p* < .001, Ƞ_p_^2^ = 0.64, Ƞ_G_^2^ = 0.30), EPA (*L*(41) = 26.445, *p* < .001, Ƞ_p_^2^ = 0.63, Ƞ_G_^2^ = 0.30), DHA (*L*(41) = 24.436, *p* < .001, Ƞ_p_^2^ = 0.56, Ƞ_G_^2^ = 0.23), omega-3 FAs (*L*(41) = 25.707, *p* < .001, Ƞ_p_^2^ = 0.61, Ƞ_G_^2^ = 0.28), omega-6 FAs (*L*(41) = 15.908, p < .001, Ƞ_p_^2^ = 0.36, Ƞ_G_^2^ = 0.19), and oleic acid (*L*(41) = 7.954, *p* = .015, Ƞ_p_^2^ = 0.09, Ƞ_G_^2^ = 0.02) (Table 9). Friedman test showed significant effects for all assessed blood parameters for the fish oil intake group, while for the olive oil intake group EPA showed a significant effect (Table 10). In the fish oil intake group, post-hoc test resulted in significant increases for omega-3 FAs (pre to during: z = − 4.824, p < .001, r = 0.74, pre to post: z = − 5.126, p < .001, r = 0.79), omega-3 index (pre to during: z = − 5.126, p < .001, r = 0.79, pre to post: z = − 4.824, p < .001, r = 0.74), EPA (pre to during: z = − 4.673, p < .001, r = 0.72, pre to post: z = − 5.276, p < .001, r = 0.81), DHA (pre to during: z = − 5.126, p < .001, r = 0.79, pre to post: z = − 4.824, p < .001, r = 0.74) and in a significant decrease for omega-6 FAs (pre to during: z = 4.221, p < .001, r = 0.65, pre to post: z = 5.276, p < .001, r = 0.81). For post-hoc test, no significant effects were found for the oleic acid in the fish oil intake group and for EPA in the olive oil intake group.

**Table 9:** Time × group interaction effects of repeated measures Puri & Sen-analysis of ranked data for blood values

	r^2^	L	p	Ƞ_p_^2^	Ƞ_G_^2^
Omega-3 Index	0.667	27.349	< .001*	0.64	0.30
EPA	0.645	26.445	< .001*	0.63	0.30
DHA	0.596	24.436	< .001*	0.56	0.23
Omega-3 fatty acids	0.627	25.707	< .001*	0.61	0.28
Omega-6 fatty acids	0.388	15.908	< .001*	0.36	0.19
Oleic acid	0.194	7.954	.015*	0.09	0.02

N = 42; fish oil and exergame *N* = 22 and olive oil and exergame N = 20. *p < .05, η_p_^2^: effect size. η_G_^2^: effect size. *DHA* Docosahexaenoic acid, *EPA* Eicosapentaenoic acid

**Table 10:** Pre-, during-, and post-measurement values of fatty acids

	Pre	During	Post	χ^2^	df	p
Omega-3 index						
Fish oil	4.83 (4.17; 5.49)	10.37 (9.00; 11.22)	9.72 (8.92; 11.58)	33.091	2	< .001*
Olive oil	5.47 (4.49; 6.19)	5.76 (5.03; 6.14)	4.99 (4.65; 5.66)	5.700	2	.058
Eicosapentaenoic acid						
Fish oil	0.70 (0.53; 0.90)	3.91 (2.79; 4.61)	3.83 (2.93; 4.80)	33.364	2	< .001*
Olive oil	0.74 (0.64; 0.94)	0.89 (0.67; 0.99)	0.73 (0.58; 0.94)	6.152	2	.046*
Docosahexaenoic acid						
Fish oil	4.08 (3.44; 4.72)	6.34 (5.95; 6.80)	6.32 (5.53; 6.69)	33.099	2	< .001*
Olive oil	4.90 (3.76; 5.22)	4.69 (4.09; 5.25)	4.27 (3.96; 4.66)	3.455	2	.178
Omega-3 fatty acids						
Fish oil	7.76 (6.60; 8.22)	14.93 (13.13; 15.79)	14.28 (12.79; 16.76)	33.091	2	< .001*
Olive oil	7.99 (7.01; 8.81)	8.22 (7.51; 9.10)	7.53 (6.88; 8.43)	2.500	2	.287
Omega-6 fatty acids						
Fish oil	32.32 (31.26; 33.31)	27.71 (26.14; 28.68)	27.10 (25.91; 27.62)	31.182	2	< .001*
Olive oil	32.54 (30.89; 33.57)	33.02 (31.70; 34.19)	33.75 (30.69; 34.74)	0.900	2	.638
Oleic acid						
Fish oil	16.08 (15.73; 17.61)	15.79 (15.08; 16.56)	16.11 (14.49; 16.96)	6.818	2	.033*
Olive oil	15.98 (15.30; 16.97)	16.19 (15.85; 17.48)	16.40 (15.78; 17.29)	3.900	2	.142

N = 42; fish oil and exergame N = 22 and olive oil and exergame N = 20. Data are median values (interquartile ranges) as indicated. Each listed fatty acid is expressed as their percentage [%] of all the fatty acids (100%) Omega-3 index is defined as the percentage of EPA and DHA in the red cell membrane, with the remaining FA building up to 100% [76]. p-values were calculated using Friedman test. *p < .05

## Discussion

The aim of this study was to investigate whether the known positive effects of exergame training can be enhanced by adding omega-3 FA supplementation. We hypothesized that the combination of exergame training and omega-3 FAs would differently affect neuronal system levels in the elderly brain compared to exergame training alone. Based on previous studies, we assumed that exergame training has positive effects on the elderly brain [37, 38]. Furthermore, previous studies showed that omega-3 FAs have positive effects on the elderly brain [85]. Although our results confirmed previous findings [27, 37–39] by showing overall improvements in some of the outcomes (time main effects), the results showed no significant time × group interaction effects in any of the primary and secondary parameters. For the blood values, significant time × group interaction effects were measured. The fish oil intake group showed a significant increase of the omega-3 FAs. This increase indicates that the participants adhered to their intake schedule which led to the increased omega-3 FA levels within the first 16 weeks. However, this increase did not lead to any additional benefits in chosen outcomes due to adding fish oil to the exergame intervention. One reason might be that the exergame training acted as the main factor evoking effects while omega-3 FAs played a subordinate role. This assumption would be in line with a recently published pilot study where the combined approach of aerobic exercise and cognitive stimulation with omega-3 FAs showed an effect on gray matter volume and sole omega-3 FAs intake in combination with placebo exercise in form of stretching and toning did not induce effects [86]. Another reason might be that an interplay exists but that our intervention study was not able to evoke and capture the effects. Several reasons may be given for this explanation. The following sections discuss possible shortcomings of our intervention design and measurement methods that might have influenced the study outcomes.

Aspects of the study design that may explain the lack of a noticeable interplay between exergame training and omega-3 FAs relate to the study population and to their intake dose and period. The selected older adults were quite fit and healthy elderly who had to be able to come to the study location by themselves. As all the participants showed a rather low level of omega-3 FAs at baseline, a huge potential existed to increase omega-3 FAs levels. The fish oil intake group showed a significant increase of the omega-3 FAs including DHA and EPA comparing pre vs post blood sample values. For fish oil supplementation, the intake duration of 16 weeks was long enough to reach a steady state condition [88–90] and the intake amount was high enough to trigger a significant increase of the omega-3 FAs values in the blood. For the omega-3 index, all the participants within the fish oil group adapted from a undesirable level of less than 4% or an intermediate-risk zone of 4–8% to a cardio protective level of 8% or higher [91] after 16 weeks. We concluded that all the participants responded well to the fish oil supplementation. From week 17 to 26, we noticed a steady state of the values while four participants showed a slight decrease of the omega-3 index to an intermediate risk zone (ranging from 5.15 to 7.81%). Even though the intake amount of 2.9 g omega-3 FAs per day seems to be an appropriate level, an individual adapted intake level might be even more promising because of genetic heterogeneity [92]. The expectation was that these increased blood levels would enhance the effects of exergame training. However, we cannot directly link the blood value course to the integration and implementation of omega-3 FAs into the neuronal system as the efficacy is limited to use blood fatty acid levels as a surrogate biomarker for central nervous system levels [93]. Therefore, the supplementation period of 16 weeks was long enough to reach a steady state level in the red blood cells of our study population, but we don’t know if the time period was also long enough to trigger an implementation effect into the brain cells and metabolism.

Another factor that might have influenced the interplay is the composition of the fish oil. The used fish oil contained DHA and EPA. However, the amount of EPA was higher than DHA. The brain contains high levels of DHA, but low levels of EPA [94]. DHA is the component that is quantitatively the most important omega-3 FAs in the brain, having unique and indispensable functions in the neuronal membrane, and in turn has positive effects on the brain [85, 93]. EPA has independent effects, particularly in regards to the respective anti-inflammatory mediators [93]. In rats, EPA and DHA increased neurite outgrowth in the development stages, nonetheless only DHA triggered positive effects in the tissue of aged rats [93]. Finally, studies indicated that the greatest benefits may be with DHA supplementation in non-cognitively impaired older people [93]. Considering these potential shortcomings, we propose that future studies should choose the fish oil composition according to their expected effects. In our study, it can be hypothesized that a higher amount of DHA might have evoked stronger effects. It seems fair to state that a better understanding of the roles of DHA and EPA to support brain health and protection is needed [93].

A further effect limiting factor could be related to the selected placebo, olive oil. We cannot exclude that the participants could profit from the olive oil as olive oil contains some effective components as well; for example, oleic acid [95]. However, the focus of this study was on the effects of omega-3 FAs and the olive oil group showed no significant increase of the omega-3 FAs during the intervention. Therefore, we concluded that the effects evoked by omega-3 FAs were minimal in the olive oil intake group. For placebo, future studies could use a fish oil supplement with a different amount of DHA and EPA as the active examined supplement depending on the intended effects.

Some methodological aspects of the selected measurements might have also limited the possibilities to capture enhanced effects. To assess omega-3 FAs values, we were bound to blood sample analysis. As mentioned before, the efficacy to use blood fatty acid levels as a surrogate biomarker for central nervous system levels is limited [93]. Moreover, it might be that our assessments, like measuring RLP, executive functions, and spatio-temporal gait parameters, were not sensitive enough to catch any effects at that stage. Neuroimaging methods might have provided more informative results than our more behavioral focused assessments. Furthermore, the used neuroimaging method TMS was probably too focused on the motor cortex only and not able to assess other brain areas. TMS measurement was limited to measure corticospinal excitability from the motor cortex to the right leg muscle (*M. tibialis* anterior). We recommend, therefore, using brain imaging methods and protocols that are not restricted to a certain brain region. A recent systematic review of Tian and colleagues, that mapped relevant brain areas for gait variability, showed that several brain regions are important for gait performance [96], and should, therefore, be considered in addition to the motor cortex. Brain imaging methods, e.g. magnetic resonance imaging (MRI) and positron emission tomography (PET), have the possibility to measure changes of grey and white matters as well as metabolic processes that might be better indicators for neuroplastic changes. A recent study, that also examined a combined approach including omega-3 FAs, stated that gray matter volume measurement might be more sensitive than behavioral outcomes to detect differences between a combined versus a single intervention [86]. Furthermore, these imaging methods would allow the measurement of several brain areas and would be able reaching deeper-located brain regions, e.g. the hippocampus. Nevertheless, it remains open if our measurement methods were not sensitive enough to catch an effect or whether our study procedure was not designed appropriately enough to trigger an evident result.

### Limitations

Some limitations of this study were already mentioned in the discussion section. In this section, we mention additional limitations that are not directly linked to the discussion section. Consideration of baseline fitness level as well as the change of the fitness level after the exergame training might have provided more information that could be used for the result discussion. Moreover, at the end of the intervention, we did not ask the participants about their assumption which nutritional supplementation they took during the intervention. Olive oil as placebo was chosen as it was the most similar to fish oil regarding to outer appearance and consistency. Nevertheless, this step could have substantiated the double-blind design. For the EEG measurements we used, some issues related to our measurement protocol might limit the measurement interpretation. The analysis showed a great range of latencies in the observed peaks. For example, the latencies (mean ± standard deviation) of the negative peak after response onset were for the fish oil and exergame group pre: Fp1 420.40 ms ± 243.13 ms, Fp2 385.60 ms ± 213.71 ms and post: Fp1 464.53 ms ± 219.58 ms, Fp2 501.26 ms ± 238.12 ms and for the olive oil and exergame group pre: Fp1 518.00 ms ± 254.31 ms, Fp2 514.00 ms ± 250.53 ms, post: Fp1 423.76 ms ± 231.23 ms, Fp2 431.13 ms ± 261.72 ms. It cannot be ruled out that differences in EEG cap positioning during the different measurement events are, at least in part, responsible for this observed variability. Nevertheless, the RLP shape was evident for the included participants, while in a few participants the RLP appeared at a later time point. Another reason for the shift in time might be due to a technical problem. Since the EEG activity was recorded using wireless signal transmission, it can be speculated that the signal transmission was slightly delayed in a few participants. Nevertheless, during the experiments other electrical devices were switched off to minimize interference. Furthermore, the randomized study design can be considered most optimal for controlling factors related to measurement issues.

## Conclusions

This study did not show that omega-3 FAs could enhance the effects of exergame training neither on aspects of the neuronal system levels in the elderly brain nor on executive functions and gait performance. To trigger possible additional beneficial effects of nutritional supplementations, future studies should bear in mind aspects of study design including study duration, individual nutritional supplementation dose, composition of the omega-3 FAs supplementation (DHA and EPA), and placebo. Furthermore, future studies should consider neuroimaging methods (e.g. MRI and PET) that might be more sensitive to assess brain adaptations at an early stage in the plasticity process. Thus, future studies should be aware of several aspects running a combinatory study that includes omega-3 FA supplementation according to their expected effects.

## Abbreviations

DHA: Docosahexaenoic acid
DTC: Dual-task cost
EDTA: Ethylenediaminetetraacetic acid
EEG: Electroencephalography
EF: Executive function
EPA: Eicosapentaenoic acid
FAs: Fatty acids
FES-I: Falls efficacy scale international
FITT: Frequency, intensity, type, and time
GDS: Geriatric depression scale
LCPUFA: Long chain polyunsaturated fatty acid
MEP: Motor evoked potential
MMSE: Mini mental state examination
MRI: Magnetic resonance imaging
NS: Nutritional supplementation
PE: Physical exercise
PET: Positron emission tomography
PFC: Prefrontal cortex
RBC: Red blood cells
RC: Recruitment curve
RLP: Response-locked potential
RMT: Resting motor threshold
TA: Tibialis anterior
TAP: Test for attentional performance
TMS: Transcranial magnetic stimulation
VG: Video game
